# Everybody's Page

**Published:** 1888-05-12

**Authors:** 


### Everybody's Page.

It is stated that £70,000 to £80,000 is paid annually in London for water sold as milk.

It is interesting to know that the poison of the cobra, although it retains its virulent properties in the dark, loses them after exposure to the rays of the sun.

Diseases of the ear frequently cause headache distinctly confined to the temple and top of the head, accompanied by pain in the ear and disturbances of hearing. Affections of the tonsils often produce headaches in precisely the same situation.

It has been proved beyond doubt that melancholics, before the days of forcible feeding, could live for over forty days without food; while many men, if they can get a supply of water, can live, and have lived, from twenty to twenty-one days.

An ordinary wind blows at the rate of six to twelve miles an hour, and such would be unbearable in a room where people were sitting. A current of air going at the rate of a mile an hour is equal to one and a-half feet a second, and such a current is not perceptible to the senses. When the air is flowing at two miles an hour, or three feet per second, then the draught begins to be observed.

Pliny, in the first century, in his famous work on natural history, distinctly refers to the cleansing body obtained from tallow and wood-ash ley, and even states that the best materials for its formation are goats' fat and the ley from the ashes of the beech tree. Such a combination would undoubtedly form soap. Further, in the ruins of Pompeii, which was overwhelmed by the eruption of Vesuvius in the year 79, there is found a thoroughly equipped soap work, so we may look on this aid to cleanliness as one of the most ancient adjuncts of civilization.

In workrooms and schools the light ought to be sufficient to allow of the occupation being prosecuted without straining the eyes of the workers. It is better to have the window high rather than low, so that the light may fall above the eyes. The pupil or workman should be so placed that he will not be in his own light, and so that when the eyes are raised from the work they will not be strained by a full glare of light, but be rested by falling upon something in comparative shade. For school children who write and for several trades it is best to have the light entering at the left hand side, and above the level of the eye. Children should not be allowed to study by twilight, nor sprawling on the hearthrug to endeavour to read by firelight. Such straining tends to induce short-sightedness.

It was greatly by observation of the anatomical peculiarities of the teeth of extinct animals, that Cuvier made those discoveries which tended to throw so much light on the subject of palæontology. By the form and appearance of any tooth found imbedded in the Montmartre quarries, near Paris, he was enabled to tell the form of jaw and head proper to the animal to which such tooth belonged; that being ascertained, the construction of the spinal column, and other bones consistent with such a character of skull, was indicated and made out. The extremities were, in like manner, prefigured in accordance with the habits and necessities implied in the conformation of those parts already known; and more strange than all, it was found that the animal thus prophetically framed and put together in the mind of the naturalist—merely from the indications afforded by a tooth—was confirmed as exactly representing the configuration of the creature to which such tooth belonged, by the subsequent discovery of the rest of its skeleton.

When a man has to go through heavy physical labour he requires and can digest a much larger amount of fat than when engaged in sedentary work. Cockles have become in times of famine a source of support, and cooked with milk and bread crumbs should be really valuable as a food.

The size of the animal seems to be important in respect to longevity. The longest livers are the whale and the elephant, 300 and 150 years respectively. While the ox lives to about sixteen, the sheep seldom reaches ten, the dog twenty, the cat between six and ten. Small animals such as mice. and smaller still, insects, live a very short time.

By statistics we learn that drapers die at a rate of 108, compared with 76 of grocers, and we also learn that this excess of deaths, in a class of men who live, as a rule, under similar social conditions to the grocers, is due to asthma and other pulmonary complaints, and are thereby pointed to the cause—close, ill-ventilated shops, having an atmosphere laden with fluff and dust, and windows and doors blocked with goods.

Badly made coal gas contains 20 or 30 per cent. of carbonic oxide, a substance which acts as a deadly poison by taking the place of the health giving oxygen in the red blood corpuscles, and forming a compound with a substance called hæmoglobin which they contain; this compound of carbonic oxide and hæmoglobin is with difficulty decomposed by oxygen. If, therefore, a large portion of the red blood globules are occupied by carbonic oxide, and thus rendered useless as oxygen carriers, we may die from asphyxia.

It is very doubtful economy to overstrain thews and muscles, it is very false economy to overstrain brain cells, for on the action of these cells the proper use of the muscles depends. When a man has to concentrate his attention his brain cells are actively at work—work implies loss of tissue and constantly diminishing functional activity; therefore concentration of attention is limited. This is practically admitted in certain services—*e.g.*, the naval and military—in the former, a man's time or turn at the wheel does not exceed two hours, and in the latter the sentry holds his post for the same term.

A fresh herring is said to supply the largest amount of nutritious animal food that can be obtained at the smallest cost. A dinner of fresh herring and potatoes furnishes all the essentials of food: Nitrogen to repair the tissues of the body, starch and fat to keep the body warm and able to work, and potash or salts, found as well as starch in the fresh vegetable the potato, to keep the blood pure and healthy. Fresh herrings may be either boiled or fried, or split open and broiled; the custom on the Clyde is to split two herrings up, clean, bone, rub with fat, and skewer them together, the skins outside, and grill above or before the fire, turning frequently.

"Where sleep the dead, who sink to rest by all their country's wishes blest?" asks Collins, and then, in answer to his question, pictures them as buried with all honours, and mourned for ever by their compatriots. Collins did not live till the time of the Russo-Turkish War, or he would have been forced to alter his poem. The bodies of those who fell in this campaign have gone to store the cellars of those artists of France and Germany who prepare skeletons, by boiling and bleaching, for medical students. A ghastly end for a brave career, and with all our respect for science we cannot help wishing that those who fall in battle had a more peaceful resting-place.

